# Protocol for a systematic review of the efficacy of epidermal grafting for wound healing

**DOI:** 10.1186/s13643-016-0268-7

**Published:** 2016-06-03

**Authors:** Muholan Kanapathy, Oliver J Smith, Nadine Hachach-Haram, Nicola Bystrzonowski, Afshin Mosahebi, Toby Richards

**Affiliations:** Division of Surgery and Interventional Science, University College London, London, UK; Royal Free Hospital Wound Healing Group, Department of Plastic and Reconstructive Surgery, Royal Free Hospital, London, UK

**Keywords:** Epidermal graft, Skin graft, Wound healing, Systematic review

## Abstract

**Background:**

Autologous skin grafting is an important modality for wound coverage; however, it can result in donor site morbidity. Epidermal grafting is an emerging option to overcome this challenge. Furthermore, it can be done in an outpatient setting with minimal or no pain. To date, the evidence on the efficacy of this technique for wound healing has yet to be outlined. We aim to synthesise the current evidence on epidermal grafting for wound healing to establish the efficacy of this technique.

**Methods/design:**

We will conduct a comprehensive search in the MEDLINE, EMBASE, and CENTRAL databases (up to May 2016) to identify studies on epidermal grafting for wound healing. We will include any primary studies (excluding case reports or case series lesser than three patients) or systematic reviews of such studies to assess the outcome of epidermal grafting for wound healing either on its own or compared to other methods. The expected primary outcome measures are the efficacy of epidermal grafting for wound healing (measured by the proportion of wounds healed at 6 weeks) and the mean wound-healing time (time for complete re-epithelialisation). Secondary outcome measures are the mean donor site-healing time, need for anaesthesia, costs associated with resource use, health-related quality of life, and proportion of patients with adverse event. Subgroup analysis will be performed for the proportions of wounds healed based on wound aetiology.

**Discussion:**

This is a timely systematic review, and the finding of this systematic review is expected to guide research and clinical practice aimed at improving wound care.

**Systematic review registration:**

PROSPERO CRD42016033051

**Electronic supplementary material:**

The online version of this article (doi:10.1186/s13643-016-0268-7) contains supplementary material, which is available to authorized users.

## Introduction

Autologous skin grafting is an important modality for wound coverage [1]. It can be classified based on the thickness of the harvested skin, namely, full-thickness skin graft (FTSG), split-thickness skin graft (SSG), and epidermal graft (EG) [2, 3].

FTSG consists of the epidermis and the entire dermis of the skin. FTSG is harvested by surgical excision, and the donor site requires primary closure. Thus, only selected areas with sufficient skin laxity are suitable for skin harvest, limiting this option for the coverage of small areas only [1]. Conversely, SSG involves the excision of the epidermis and part of the dermis, using an electric air dermatome, leaving behind the reticular dermis in the donor site enabling the skin to heal by secondary intention [1]. It is the commonest form of autologous skin grafting performed and can be meshed to cover a wide surface area [1]. However, the donor site becomes a second, often painful wound, which may take more time to heal than the graft site itself and holds the risk of infection and scarring [4]. Both the FTSG and SSG often require hospital admission, even as a day case, anaesthesia, and a period of immobility for some patients.

Epidermal grafting, on the other hand, is an emerging and promising option to overcome these challenges. EG for wound healing is relatively recent compared to FTSG and SSG, which have been performed since the eighteenth century [2, 5, 6]. EG involves harvesting only the epidermal layer of the skin from the donor site by applying continuous negative pressure on the normal skin to raise a blister. The roof of the blister, which is the epidermis, is then excised and transferred onto the wound. As the dermis in the donor site remains untouched, the skin regenerates itself without a scar. This procedure is also often painless as the pain fibres in the dermis are unstimulated, allowing autologous skin grafting in the outpatient setting. The use of EG for treating wounds has been on the rise of late, with several recent publications in the last couple of years, as it allows autologous skin grafting in the outpatient setting without anaesthesia and with minimal donor site morbidity [3, 7–11].

Although there are several reports on the successful wound healing with epidermal grafting, to date, the evidence on the efficacy of this technique for wound healing has yet to be outlined. A scoping search was undertaken in the MEDLINE (OvidSP), EMBASE (OvidSP), and Cochrane Central Register of Controlled Trials (CENTRAL) databases to identify existing systematic reviews and to gauge the volume of any primary studies on the topic. No existing systematic reviews were identified, but there are a number of primary studies on epidermal grafting for wound healing. It is timely that the evidence on the efficacy of this technique is assessed to guide clinical decision-making and facilitate future intervention research.

## Methods/design

### Objective

This systematic review synthesises the current evidence on epidermal grafting for wound healing to establish the efficacy of this technique in the clinical setting by measuring the proportion of wounds healed and the mean wound-healing time (time for complete re-epithelialisation).

### General methods

This protocol has been registered with the PROSPERO international prospective register of systematic reviews (registration number, CRD42016033051) and was reported adhering to the Preferred Reporting Items for Systematic Review and Meta-Analysis Protocols (PRISMA-P) 2015 statement (see Additional file 1) [12]. The final review will be reported following the PRISMA statement. In the event of no randomised controlled trial (RCT) available to be included, the systematic review will be reported according to the Meta-analysis Of Observational Studies in Epidemiology (MOOSE) guidelines [13].

### Search strategies

We will search the MEDLINE (OvidSP), EMBASE (OvidSP), and CENTRAL databases from 1946 to identify studies of relevance to this review. Publicly available trial registers (ClinicalTrials.Gov and WHO International Clinical Trials Registry Platform) will be searched for all trials. The reference list of all articles included will be cross-checked for further articles of relevance.

The search strategy will use a combination of text word and Medical Subject Headings (MeSH) terms relating to the use of epidermal graft in treating wounds. There will be no restriction by study design or outcomes as the research questions are broad. No date, language, or publication restriction will be applied. A sample search strategy for MEDLINE (OvidSP) is shown, and a similar strategy will be adapted for use in other databases.

1. [epidermal graft*] OR [blister graft*] OR [suction blister*] OR [suction graft*]
2. Epidermis/su, tr [Surgery, Transplantation]
3. [1] or [2]

### Selection criteria

All human studies related to epidermal grafting for treating wounds will be included.

### Study design

We will include any primary studies (excluding case reports or case series lesser than three patients), or systematic reviews of such studies, assessing the outcome of epidermal grafting for wound healing either on its own or compared to other methods.

### Type of participants

The participants are adult patients (18 years and above) with wounds of any size and aetiology treated by epidermal grafting.

### Setting

Studies performed in any clinical setting will be included.

### Intervention

This will include any epidermal graft-harvesting techniques which involve creating suction blisters using negative pressure. Information about the harvest methods such as the amount of negative pressure generated, harvest device, harvest time, and dressing used after grafting will be documented.

### Comparator

Studies comparing epidermal grafting to wounds managed by dressings only will be included in this review.

### Outcome measures

The primary outcome measures are the proportions of wounds with complete healing at 6 weeks and the mean wound-healing time (time for complete re-epithelialisation). Secondary outcome measures are the mean donor site-healing time, need for anaesthesia, economic evaluation based on the cost associated with resource use, health-related quality of life, and proportion of patients with adverse event. Subgroup analysis will be performed for the proportions of wounds with complete healing based on the wound aetiology.

### Exclusion criteria

The exclusion criteria are as follows: case series of less than three cases; studies describing the use of epidermal grafting in skin pigmentation disorder such as vitiligo; and studies describing only the harvest technique without treatment outcome.

### Study selection and data management

Study selection will be conducted in a two-stage process. The titles and abstracts will initially be screened by two reviewers, using pre-specified screening criteria, for potential eligibility after excluding duplicate records. Next, studies identified as relevant will undergo full-text review by both reviewers. Any discrepancies between the reviewers will be resolved by discussion or by referral to a third reviewer. Any relevant non-English language articles will be translated where necessary. The search results, including abstracts, full-text articles, and record of the reviewer’s decisions, including reasons for exclusion, will be recorded in Endnote X7.

### Data extraction

The data from all full-text articles accepted for final analysis will be independently retrieved by two authors using a standardised data extraction form. Any disagreements and differences will be resolved by discussion or referral to a third reviewer. Primary study authors will be contacted if further information is needed or some data are missing.

The following data will be extracted:

- Study characteristics (authors, year of publication, country of publication, study design)
- Patient demography (number of studied subjects, sex, mean age, comorbidity, number of wounds treated)
- Wound characteristics (wound aetiology, mean wound duration, mean wound size, pre-grafting wound quality)
- Characteristics of intervention and control group including the EG harvest technique (device used, amount of negative pressure generated, duration of harvest), use of anaesthesia, donor site dressing, and wound dressing
- Outcomes (wound-healing time, number and type of wounds with 100 % re-epithelialisation at 6 weeks, number and type of wounds with 50–99 % healing at 6 weeks, number and type of wounds with lesser than 50 % healing at 6 weeks, donor site-healing time)
- Length of follow-up
- Cost for EG and dressings only for wound management
- Health-related quality of life in patients managed with EG and dressings only
- Complications or adverse events (incidence of device-related adverse events (DAEs) and the incidence of wound-related adverse events (WAEs) occurring within the study duration)
- Statistical analysis model utilised

### Assessment of risk of bias of included studies

Included studies will be critically appraised for methodological quality and risk of bias by two review authors independently. Discrepancies will be resolved through consensus or referral to a third reviewer if necessary. The included studies are expected to all be observational studies, with no RCTs. Should there be any RCTs, they will be assessed according to the Cochrane Collaboration Risk of Bias Assessment Tool [14]. The observational cohort studies will be assessed using the Cochrane Risk of Bias Assessment Tool for Non-Randomized Studies of Interventions (ACROBAT-NRSI) [15]. This will evaluate the risk of bias due to confounding, selection, measurement, and interpretation. The quality of reporting will be assessed using the Strengthening the Reporting of Observational Studies in Epidemiology (STROBE) checklist [16].

### Data analysis and synthesis

The main outcome measure of the included studies will be the pooled estimate of the proportions of wounds healed at 6 weeks, the mean wound-healing time, and the mean donor site-healing time with corresponding 95 % confidence intervals. Meta-analysis will be performed should a sufficient number of studies with consistent characteristics be found in terms of study design and outcome reporting. We will explore the sources of potential clinical and methodological heterogeneity based on the study design, population, intervention, and comparator characteristics and outcomes. Statistical heterogeneity will be assessed using the chi-square test and quantified with the *I*^2^ statistic. The thresholds for interpretation of *I*^2^ will be in accordance with the definitions presented in the Cochrane Handbook for Systematic Reviews of Interventions [17].

Narrative synthesis will be performed in the event that the meta-analysis is not appropriate. The narrative synthesis will be grouped by the outcome of interest. As several different EG harvest devices are expected to be used, the difference between the devices will likely be synthesised narratively. We also expect wounds from various aetiologies to be treated. The wounds will be broadly classified into acute (<3 months in duration) and chronic (≥3 months in duration), and the difference in outcome will then be synthesised narratively.

Data synthesis will be performed using Review Manager 5.3 provided by The Cochrane Collaboration [17]. Should no RCT be available, a meta-analysis of observational study will be performed using StatsDirect Statistical software (StatsDirect statistical software, version 2.8.0; StatsDirect, Altrincham, UK). The quality of evidence will be rated using the Grading of Recommendations Assessment, Development and Evaluation (GRADE) approach.

## Discussion

In this review, we aim to determine the efficacy of epidermal grafting for wound healing by evaluating the overall success rate of this technique for wound healing and the mean wound and donor site-healing time which is currently unclear. As epidermal grafting is now emerging as a potential alternative to the more invasive traditional techniques (FTSG and SSG), it is important that the current literature on EG be evaluated to determine whether its efficacy for wound healing is comparable or better than the current treatment options. To our knowledge, this is the first systematic review to outline the efficacy of epidermal grafting for wound healing. The finding of this systematic review is expected to guide research and clinical practice aimed at improving wound care. In particular, if epidermal grafting is efficacious for wound healing, it may provide a cheaper and less invasive alternative to traditional methods. It may also provide better donor site outcomes and improved patient satisfaction due to less scarring and pain.

## Limitations

We expect the sensitivity of our search to be limited by the lack of MeSH terms for epidermal grafting. We have included a wide range of text word combinations to overcome this.

## Abbreviations

ACROBAT-NRSI, A Cochrane Risk of Bias Assessment Tool for Non-Randomized Studies of Interventions; CENTRAL, Cochrane Central Register of Controlled Trials; EG, epidermal graft; FTSG, full-thickness skin graft; MeSH, Medical Subject Headings; MOOSE, Meta-analysis Of Observational Studies in Epidemiology; PRISMA-P, Preferred Reporting Items for Systematic review and Meta-Analysis Protocols; RCT, randomised controlled trial; SSG, split-thickness skin graft; STROBE, Strengthening the Reporting of Observational Studies in Epidemiology

## Additional file

Additional file 1: PRISMA-P checklist. (DOC 83 kb)
